# Mitochondrial responses to brain death in solid organ transplant

**DOI:** 10.3389/frtra.2023.1082227

**Published:** 2023-03-21

**Authors:** Meredith E. Taylor, Dinesh Jaishankar, Jessie W. Ho, Hasan B. Alam, Ankit Bharat, Satish N. Nadig

**Affiliations:** ^1^ Department of Surgery, Feinberg School of Medicine, Chicago, IL, United States; ^2^ Division of Organ Transplant and Comprehensive Transplant Center, Feinberg School of Medicine, Chicago, IL, United States; ^3^ Division of Thoracic Surgery and Pulmonary and Critical Care Medicine, Feinberg School of Medicine, Chicago, IL, United States; ^4^ Department of Microbiology-Immunology, and Pediatrics, Comprehensive Transplant Center, Feinberg School of Medicine, Northwestern University, Chicago, IL, United States; ^5^ Simpson Querrey Institute, Northwestern University, Chicago, IL, United States

**Keywords:** solid organ transplantation, mitochondria, brain dead (BD) organ donor, brain death, mitochondrial therapeutics, immunometabolism, mitochondrial dynamics

## Abstract

Mitochondrial dynamics are central to the pathophysiology of cellular damage and inflammatory responses. In the context of solid organ transplantation, mitochondria are implicated in immune activation in donor organs that occurs after brain death, as they are critical to the regulation of cellular stress response, cell death, and display energetic adaptations through the adjustment of respiratory capacity depending on the cellular milieu. Mitochondrial damage activates mitochondrial systems of fission, fusion, biogenesis, and mitochondrial autophagy, or mitophagy. The mechanistic pathways as well as therapies targeting mitochondrial physiology have been studied as plausible ways to mitigate the negative effects of brain death on donor organs, though there is no summative evaluation of the multiple efforts across the field. This mini-review aims to discuss the interplay of donor brain death, mitochondrial dynamics, and impact on allograft function as it pertains to heart, lung, liver, and kidney transplants.

## Introduction

Solid organ transplantation has significantly improved the survival and quality of life of patients with end-stage organ failure. However, there is a discrepancy between the number of organs needed versus the number of organs available for transplantation. This has led to the utilization of organs from deceased patients (1–3). The United Network of Organ Sharing (UNOS) reported that 13,861 individuals in the United States became deceased donors for organ transplants in 2021, an increase for the eleventh year in a row (1). Despite being the leading resource for organs in those needing lifesaving transplants, metabolic changes that occur due to BD and in the subsequent stages of organ procurement, storage, and implantation results in inflammatory responses in the donor organs that are then “primed” for immune recognition (2–4). The interplay between immune response and organ longevity is a of long-term graft viability and success in solid organ transplantation (2, 5). The opportunity to optimize graft health, usability, and longevity begin at the time, and even prior to, donor BD and continues throughout the life of the recipient. Therefore, there is a great need to optimize all viable organs from BD donors. Addressing early insults to the allograft caused by donor BD can be an effective therapeutic strategy.

Following BD, physiological homeostasis is disrupted followed by increased intracranial pressure and brainstem ischemia (3, 4, 6–8). Subsequently, catecholamine release leads to systemic vasoconstriction and decreased blood flow to peripheral organs (4). Increased circulating catecholamine levels, specifically epinephrine and norepinephrine, have been observed in both rat and porcine models of BD (9). The catecholamines have been demonstrated to mitigate multiple metabolic changes, including shifts to anaerobic metabolism and endothelial activation from shear stress caused by vasoconstriction (6). There is evidence of increased inflammatory cytokines, interleukins (IL)-6, IL-8, IL-1 beta, and tumor necrosis factor-alpha (TNF-α) in the peripheral circulation of humans measured by serum assays following BD (10–12) suggesting that BD induces a systemic inflammatory response through physiological compensatory mechanisms and metabolic disruption. Importantly, it has been previously established that traumatic brain injury (TBI), the second most common process leading to BD (13), also causes a systemic inflammatory response (3, 6, 8, 14–19). A swine model of combined TBI and hemorrhage demonstrated activation of the complement cascade immediately post-TBI through increased C5a levels (7). Post-injury, there were increased levels of the inflammatory markers IL-6 and TNF-α following the injury (7). Potential donor organs are exposed to the inflammatory environment caused by both TBI and subsequent BD, likely leading to increased graft immunogenicity and poorer transplant outcomes (6, 8, 9).

**Figure 1:**
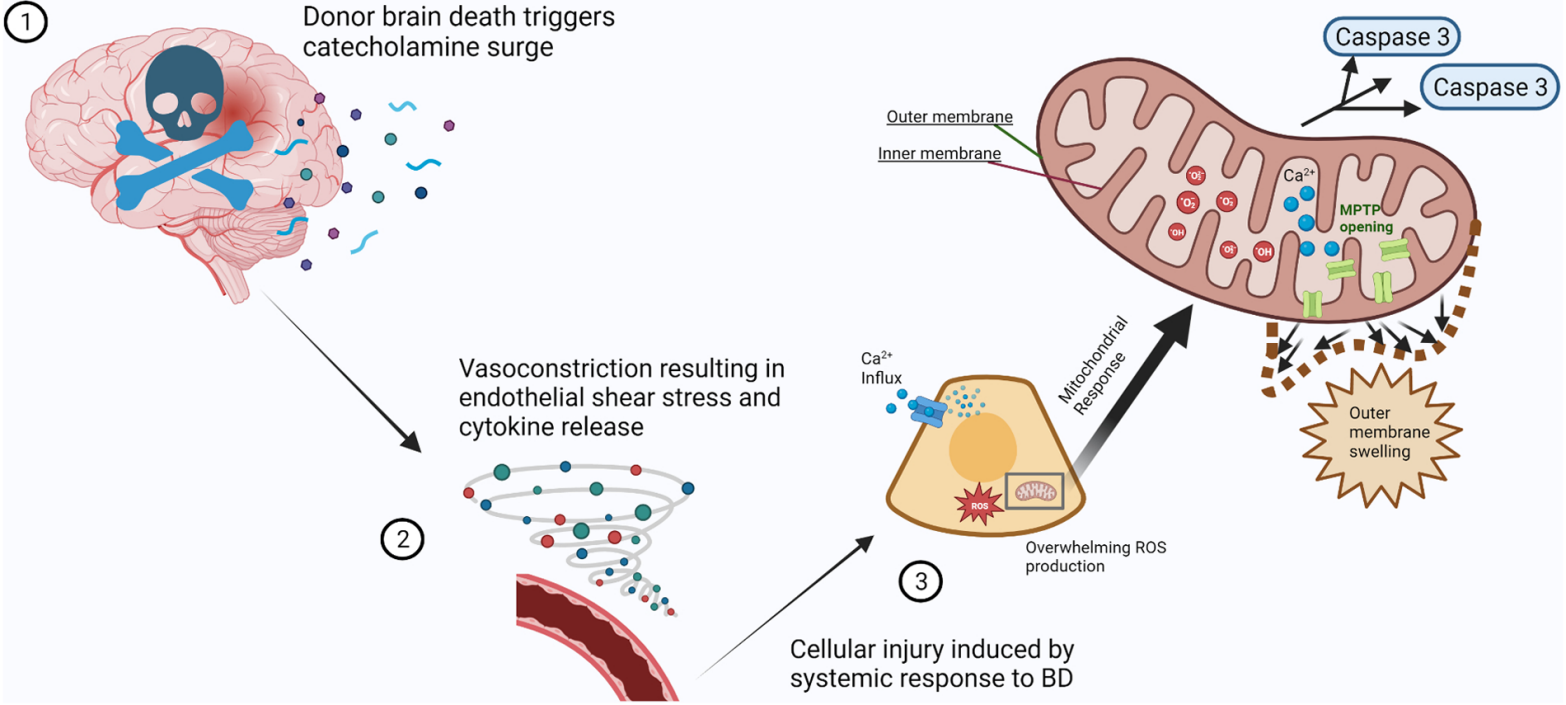
Mitochondrial response to cellular injury after profound physiological disturbances secondary to brain death. (**1**) Brain death of the organ donor results in a systemic surge in catecholamine production (spheres and curved ribbons). (**2**) Vascular response to catecholamine influx results in vasoconstriction and increased blood pressure. The resulting vascular endothelial shear stress causes cytokine release (vortex of spheres), triggering multiple inflammatory pathways. (**3**) The systemic response to brain death causes prolonged cellular stress. Mitochondrial compensation for calcium influx and neutralization of reactive oxygen species is overwhelmed (inset), causing MPTP opening, outer membrane swelling, and release of pro-apoptotic factors such as caspases. Created with BioRender.com.

Mitochondria are cellular organelles composed of an outer and inner membrane, with the latter housing the four protein complexes that make up the electron transport chain (ETC). Through the process of cellular respiration and oxidative phosphorylation, the ETC generates adenosine triphosphate (ATP), the main cellular energy source (2, 3). The production of ATP leads to the generation of reactive oxygen species (ROS) as a by-product. Under normal physiologic conditions, the ROS are kept in balance by antioxidant systems (4, 20–22). Periods of cellular stress cause an imbalance in the production of ROS by mitochondria, where the amount produced overwhelms cellular antioxidant capabilities, causing further insult to cells (2, 4, 22, 23). In addition, mitochondria play an integral role in cellular autophagy, or programmed cell death, as a response to injury (Figure 1). Mitochondria contribute to autophagy through the mitochondrial permeability transition pore (MPTP), an impartial pore on the inner membrane of mitochondria that opens under conditions associated with cellular stress, such as calcium influx, oxidant accumulation, and a decrease in nucleotides (24, 25).

Recent studies have begun to describe the mechanisms of mitochondria, mitochondrial DNA (mtDNA), and DAMP production in the inflammatory responses associated with BD organ donors (7, 26). Here, we aim to review mitochondrial mechanisms affected by BD and their implications in solid organ transplantation. This mini-review will summarize the existing literature linking BD to mitochondrial activity and inform future studies interested in pursuing therapeutics targeted to mitochondrial injury responses pre-transplant.

## The effect of brain death on allograft mitochondrial function

Trauma, TBI, and BD have been shown to activate the donor immune system and establish a pro-inflammatory environment (6–8, 27, 28) and several reports suggest that mitochondrial metabolism contribute to this process. In donor organs from BD patients, decreased ATP levels and energetic imbalances are observed and thought to be secondary to impaired oxidative metabolism and a shift between aerobic to anaerobic respiration (16, 29). An early study conducted on human BD organ donors demonstrated differences in mitochondrial metabolism when compared to healthy subjects (29). BD donors were observed to have higher plasma lactate-to-pyruvate ratios. Decreased mitochondrial respiration rates during ATP synthesis were observed using donor muscle biopsies, suggesting mitochondrial dysfunction (29). Additionally, mitochondrial activation and accumulation in injured endothelial cells of donor organs have been directly related to immunogenicity by increased T cell activation and adhesion, further demonstrating the link between early graft insults caused by donor BD, mitochondria, and immune response (3, 30). As the endothelial cell barrier is the first interface between an allograft and recipient, a murine endothelial cell model was used to demonstrate that endothelial cell treatment with extracellular mitochondria was associated with up-regulated cell surface adhesion molecules, CD54 (ICAM-1), CD106 (VCAM-1) and CD62E (E-selectin) (30). An increase in surface adhesion molecules may lead to immune activation through neutrophil, monocyte, and lymphocyte adhesion (30). Pollara et al. demonstrated that BD donor plasma has increased circulating free and membrane-bound mitochondria. They also noted a positive correlation between mtDAMPs and the inflammatory cytokines IL-6, IL-8, IL-2R, and IL-1A (3). Importantly, increased circulating mtDNA was associated with early allograft dysfunction in liver transplant recipients (3). The mitochondrial mechanisms involved in donor BD and immune response create the environment for allografts, demonstrating their importance.

While the global metabolic response of BD has commonalities across organ systems, there is value in delineating mitochondrial mechanisms given the many organ-specific differences in solid organ transplantation. Donor BD has been attributed to negative outcomes in cardiac transplantation such as primary graft dysfunction and early graft failure through the activation of immunological responses and propagation of a pro-inflammatory state (4, 20, 21, 26, 27). Constant muscular contraction of the functioning heart leads to high baseline metabolic demand and necessitates increased concentration of mitochondria in the myocardium, accounting for about 33% of the cell contents (4). It is thought that the catecholamine surge instigated by BD results in a hyperdynamic cardiac state with a depletion of energy stores and increased myocardial oxygen consumption. Catecholamines also increase ROS production through mitochondrial activation as well as an influx of mitochondrial calcium, which is postulated to lead to increased levels of cellular apoptosis, the opening of mitochondrial permeability transition pores (MPTP), and mitophagy (4). However, there have been no studies directly confirming mitochondrial dysfunction in cardiac tissue after donor BD. The pathological impact of BD on donor lungs has been well-studied, focused on two major processes occurring after TBI, neurogenic pulmonary edema and the pulmonary contribution to the systemic inflammatory response (22). The overwhelming pulmonary edema after brain injury has also been attributed to epinephrine and norepinephrine release. Recent data has shown a complementary immune contribution implicating non-classical monocytes in both murine models and patients (14). Although prior research has explored the mechanistic involvement of mitochondria in other modalities of lung injury and pathology, such as pulmonary fibrosis and the aging process (23), this pathway has not been explored in regard to BD and donor lungs and requires further study. The negative effects of BD on donor liver longevity and immunogenicity have been connected to mitochondrial dysfunction-related enzymatic pathways. Upregulated hepatocyte apoptosis in donor organs recovered after BD is thought to be one of the mechanisms contributing to poorer graft function in comparison to living donors (15, 24, 25). Using proteomics analysis and a model of BD in a rabbit, aldehyde dehydrogenase 2 (ALDH2), a mitochondrial matrix protein and protective factor against oxidative stress, was found to be downregulated in livers collected from BD animals with a parallel increase noted in hepatocyte apoptosis (15). When compared with living donation, decreased levels of ATP and renal perfusion with elevated renal injury markers (plasma urea and creatinine) were demonstrated in kidneys after BD in a rat model compared to a sham event (16). However, mitochondrial respiration measured by oxygen consumption rate in various metabolic pathways did not show a difference between the BD and sham model, possibly indicating the change in renal perfusion and oxygen delivery after BD has a greater impact than mitochondrial dysfunction (16). They did demonstrate a shift from aerobic to anaerobic metabolism, in which mitochondrial machinery is the main driver, so perhaps further study is needed to fully appreciate the mitochondrial impact.

While overall, donor BD is known to negatively impact the health of donor organs, there is a need for more focused mechanistic studies characterizing organ-specific mitochondrial changes. Given the vast differences in each organ's structure and physiology, the unique mitochondrial changes in each organ may continue to drive novel therapeutic advances in transplantation.

## Mitochondria as a therapeutic target

The majority of pharmacotherapeutics focused on BD donor treatment were originally explored as strategies targeting ischemia-reperfusion injury (IRI) in donor organs.

SP600125 (anthrapyrazolone), a c-Jun N-terminal kinase (JNK) inhibitor, was initially shown to decrease myocardial damage in IRI (31). In a model of rat BD, Guo et al. demonstrated decreased levels of cytochrome C and caspase- 3 mRNA expression with a concurrent decrease in myocardial apoptosis *via* terminal deoxynucleotidyl transferase dUTP nick end labeling (TUNEL) assay (32). In the mitochondrial pathway, JNK induces cytochrome-C release leading to caspase-3 activation (33). Caspase-3 promotes the degradation of intracellular structural substrates such as cytoskeletal proteins, targeting the cell for apoptosis (34). The study demonstrated increased levels of cytochrome C and caspase-3 expression in the untreated BD rats with a simultaneous increase in myocardial apoptosis (32). SP600125 has also been explored as a therapeutic to mitigate the effects of BD on the donor liver in a BD donor rat model (35), and similar protective effects were observed (35). BD rats treated with SP600125 also had decreased levels of aspartate aminotransferase (AST) and alanine transaminase (ALT), in comparison to BD controls, suggesting functional protection of the allograft (35). Based on the current understanding of SP600125 in a BD model, further exploration is needed as to whether the reduction in apoptosis improves long-term allograft function.

Dimethyloxalylglycine (DMOG) is a prolyl-hydroxylase inhibitor that activates hypoxia-inducible factor-1 (HIF-1) (36). Based on the concept of pre-conditioning for hypoxic and ischemic states through HIF-1 activation, DMOG therapy was chosen as a way to mitigate cardiac allograft damage induced by BD (36). Pre-treatment of BD donor rats with DMOG led to improved early systolic function, less inflammatory infiltrate, and less myocardial necrosis (36). DMOG pre-treated donors showed decreased levels of caspase-3 gene expression, indicating that a decrease in pro-apoptotic factors likely protects an allograft from a BD donor (36). Nitric oxide (NO) is another compound associated with the regulation of mitochondrial respiration and IRI protection. A rat model of renal transplant with BD donors treated with a bolus injection of sodium nitrite demonstrated decreased infiltrating inflammatory cells and better post-transplant renal function (37). While the direct impact of NO on mitochondrial function was not specifically studied, based on the observed effects of DMOG in cardiac transplant, it may be an opportunity for a future therapy targeting mitochondrial apoptosis.

Overexpression of heme oxygenase 1 (HO-1), a mediator of cytoprotective anti-inflammatory and anti-apoptotic function, has been proven as a therapeutic target in hepatic IRI (38). The effects of a known HO-1 inducer cobalt protoporphyrin (CoPP) on hepatic function after animal BD were studied in a rat model (38). Fang et al. demonstrated, HO-1 overexpression through CoPP administration was correlated with a significant decrease in AST, ALT, and apoptotic hepatocyte levels in comparison to BD controls (38). They also similarly demonstrated decreased expression of cytochrome C and caspase-3, indicating modulation of the mitochondrial apoptosis pathway. While CoPP administration has never been evaluated in humans, it may be a viable future therapy for limiting mitochondrial injury in a BD donor.

EGb761, derived from the *gingko biloba* plant, has demonstrated neuroprotection, mitochondrial protection, anti-inflammatory properties, and to have a renal protective effect in IRI (39). Therefore, it was hypothesized that EGb761 may also reduce BD-associated injury in kidneys. EGb761 was administered to rats 48 h prior to and three times following surgically induced BD (39). Kidneys from the EGb761-treated rats had decreased blood urea nitrogen (BUN), creatinine, and mitochondrial swelling when viewed *via* transmission electron microscopy (39). A reduction in macrophage infiltration on renal histology as well as decreased IL-6 and TNF-α was also demonstrated in the EGb761-treated animals (39). This study suggests a mitochondrial protective effect through EGb761 treatment of the BD donor, leading to decreased inflammation and improved organ function.

While most of the focus on mitochondrial therapy in the BD donor has been on the role of mitochondria in the apoptotic pathway, ROS generation, and the association with inflammatory markers, an additional strategy is targeting mitochondrial dynamics. In a recent study, our laboratory examined the relationship between mitochondrial dynamics and immunogenicity using a murine BD, heterotopic cardiac transplant model (21). Mitochondrial fission, fusion, and the interplay between the two mechanisms are important to maintaining mitochondrial health and function (40). The BD donor mice were infused with a combination of mitochondrial fusion promoter (M1) and fission inhibitor (Mdivi1) prior to organ procurement and transplant (21). The recipient mice of the pre-treated hearts demonstrated longer allograft survival and decreased immunogenicity with decreased production of cytotoxic proteins by T-cells (21). The model combines modulating energy metabolism and mitigation of the inflammatory cascade (21). It highlights how an optimal pre-treatment solution can target an organ's immunometabolism pre-implantation. The benefit of increasing mitochondrial fission in a BD donor has also been observed in the kidney. N-guanyl-1,7-diaminoheptane (GC7) is a competitive inhibitor specific for deoxyhypusine synthase (DHS), an enzyme active in post-translational modification of lysine (41). Using an immortalized renal proximal cell line, GC7 treatment *in vitro* showed a reversible induction of mitochondrial silencing mechanisms through suppressed expression and activity of the respiratory chain complexes (42). Pre-treatment of porcine kidneys with GC7 prior to warm ischemia and cold storage was shown to decrease apoptosis immediately post-transplantation, have earlier recovery of renal function, and reduced late interstitial fibrosis (42). The authors sought to understand whether modulating the same pathway in a porcine model of BD would yield similar results. BD was induced by slowly filling a balloon in the epidural space through a small hole drilled in the skull, followed by donor infusion of GC7, a 4-h management period, renal procurement, and transplantation (17, 43). The recipient animals were followed for 90 days post-transplant with histological analysis of the allograft upon euthanasia (17). After the 4-h management of the BD donor, those that received GC7 therapy showed a decreased generation of ROS and markers induced by BD, as well as an upregulation of the expression of mitochondrial biogenesis protein, PGC1-α (17). Their work also indicates treatment with GC7 favors mitochondrial fission through the MFN2 pathway, preserving mitochondrial integrity (17). Importantly, at 90 days post-transplant, renal structure and function were better in the pigs that received organs from BD donors treated with GC7 (17).

While the fields of cardiac, hepatic, and renal transplantation have begun to test the effects of mitochondrial pharmacotherapies for mitigating BD-associated allograft injury (Table 1), there are no current therapies for pulmonary transplantation. Thus, there is an opportunity to explore the utility of mitochondrial-targeted therapies in lung transplantation. Overall, there is a relative paucity of literature examining organ-specific mitochondrial responses in BD donor organs. Modulating mitochondrial responses to cellular stress and mitochondrial dynamics are two promising strategies for the treatment of the BD donor to preserve allograft integrity.

**Table 1:** Mitochondrial targeted therapies utilized for treatment of the BD organ donor.

Organ	Mechanism of action	Therapy	Mitochondrial effect
Heart	Prolyl-hydroxylase inhibitor, activates hypoxia-inducible factor-1 (36)	Dimethyloxalylglycine (DMOG)	Decreased caspase-3, blunts activation of mitochondrial apoptosis pathway (36)
Heart	DRP inhibitor (21, 44)	Mdivi-1	Mitophagy induction, fission inhibitor (21)
Heart	Mitofusins 1 and 2, Opa1 (21, 44)	M1	Mitochondrial fusion promoter (21)
Kidney	Antioxidant (39)	EGb761 (gingko biloba extract)	Decreased mitochondrial swelling, membrane stabilization (39)
Kidney	Competitive inhibitor of deoxyhypusine synthase (17, 41)	N-guanyl-1,7-diaminoheptane (GC7)	Decreased ROS, upregulation of mitochondrial biogenesis protein, PGC1-α, increase fission through MFN-2 pathway (17, 42)
Liver	Heme oxygenase 1 induction (38)	HO-1 inducer cobalt protoporphyrin (CoPP)	Decreased cytochrome C and caspase-3 expression, blunts activation of mitochondrial apoptosis pathway (38)

## Concluding remarks

Mitochondrial activity and their capacity for ATP synthesis make them an important mediator of cellular injury, immune activation, and cell death during each phase of organ transplantation, from donor BD to recipient implantation. The mechanistic pathways present numerous potential avenues for therapeutic targets to mitigate the negative effects of BD on organs (45–51). The current literature on mitochondrial response during BD in the setting of solid organ transplant has centered around understanding mitochondrial mechanisms and elucidating potential therapeutic targets for pharmacological intervention. While previous work has allowed for the postulation of strategies to modify apoptotic mechanisms, decrease ROS generation, and replenish mitochondria, few have allowed for actionable interventions in the clinical setting. There is also an understanding of immune activation caused by BD, endothelial injury, and its association with mitochondrial function, dysfunction, and regulation. The connection between endothelial cell barrier disruption, ROS generation, and robust immune response after donor BD suggests the existence of a *neuroendothelial axis*. Targeting mitochondrial responses at each portion of the axial dynamics is an opportunity to blunt the donor response to BD and its impact on potentially transplantable organs. Furthermore, there is a gap in our understanding of mitochondrial dynamics modification at each stage in organ transplantation and the synergistic impact of mitochondrial therapy on short and long-term donor organ viability. Specifically, there is a need to address the paucity of strategies directed at modulating the effect of donor BD on the peripheral organs at both the macro and cellular levels. The challenge will be to better understand the differences between solid organs and how each mitochondrial response to BD can be addressed through the treatment of the donor and organs. The therapeutic potential of harnessing the power of known mitochondrial therapies and mitochondrial mechanisms across the field of solid organ transplant is immense. The current body of work has illustrated multiple opportunities to address donor organ injury and immunometabolism at every stage, starting at donor BD.

## Author contributions

MT and JH participated in literature search and data analysis. HA, AB, and SN provided fundamental ideas and topic expertise for the literature review and manuscript. MT, DJ, and SN oversaw the outlining and drafting of the paper. All authors contributed to the article and approved the submitted version.
